# Aortic dissection during antiangiogenic therapy with sunitinib. A case report: Dissecção de aorta em terapia anti-angiogênica com sunitinibe. Um relato de caso

**DOI:** 10.1590/1516-3180.2013.7380002

**Published:** 2014-10-28

**Authors:** Maria Nirvana da Cruz Formiga, Marcello Ferretti Fanelli

**Affiliations:** I MD, MSc. Medical Oncologist, Hospital A. C. Camargo, São Paulo, Brazil.; II MD, MSc. Head of Department of Clinical Oncology, Hospital A. C. Camargo, São Paulo, Brazil.

**Keywords:** Antineoplastic agents, Angiogenesis inhibitors, Aortic aneurysm, Carcinoma, renal cell, Hypertension, Antineoplásicos, Inibidores da angiogênese, Aneurisma aórtico, Carcinoma de células renais, Hipertensão

## Abstract

**CONTEXT:**

Sunitinib is an antiangiogenic drug that has been approved for treating metastatic renal cancer. Its action as a tyrosine kinase inhibitor of vascular endothelial growth factor receptors (VEGFRs) and other angiogenesis receptors may lead to adverse effects such as hypertension and heart failure. However, reports in the literature on an association between sunitinib therapy and acute aortic dissection are rare.

**CASE REPORT:**

We report the case of a 68-year-old man with metastatic renal carcinoma who developed acute aortic dissection during sunitinib therapy. He had no history of hypertension or any other risk factor for aortic dissection. After aortic dissection had been diagnosed, sunitinib was withdrawn and an aortic endoprosthesis was placed. Afterwards, the patient was treated clinically with antihypertensive drugs and new therapy for renal cancer consisting of temsirolimus, an inhibitor of the mammalian target of rapamycin (mTOR) pathway.

**CONCLUSION:**

Hypertension is a common event when antiangiogenic drugs are used in oncology. However, knowledge of other severe cardiovascular events that may occur in these patients, such as acute aortic dissection, is important. Adequate control over arterial pressure and frequent monitoring of patients during the first days of antiangiogenic therapy is essential for early diagnosis of possible adverse events.

## RESUMO

**CONTEXTO:**

Sunitinibe é uma droga antiangiogênica aprovada para tratamento de câncer renal metastático. Sua ação como inibidor de tirosina quinase de receptores de fatores de crescimento do endotélio vascular (VEGFR) e de outros receptores de angiogênese pode levar a eventos adversos como hipertensão e insuficiência cardíaca. No entanto, é escassa na literatura a associação da terapia com sunitinibe e dissecção aguda de aorta.

**RELATO DE CASO:**

Relatamos o caso de um paciente do sexo masculino de 68 anos com câncer renal metastático que desenvolveu dissecção aguda de aorta durante tratamento com sunitinibe. O paciente não tinha histórico prévio de hipertensão nem outro fator de risco para dissecção de aorta. Após diagnóstico da dissecção de aorta, a droga foi suspensa e o paciente foi submetido à colocação de endoprótese na aorta, evoluindo posteriormente com controle clínico da pressão arterial e nova terapia para câncer renal com tensirolimo, um inibidor da via proteína alvo da rapamicina em mamíferos (mTOR).

**CONCLUSÕES:**

A hipertensão é um evento comum com uso de drogas antiangiogênicas na oncologia. No entanto, é importante o conhecimento de outros eventos cardiovasculares graves, como dissecção aguda de aorta, que podem ocorrer nesses pacientes. Controle adequado da pressão arterial e monitorização frequente dos pacientes nos primeiros dias de terapia antiangiogênica são essenciais para diagnóstico precoce de possíveis eventos graves.

## INTRODUCTION

Sunitinib is a tyrosine kinase inhibitor that targets several vascular endothelial growth factor and platelet-derived growth factor receptors. It is indicated as first-line therapy in metastatic renal cell carcinoma, at a dose of 50 mg given orally once daily for four weeks, in a six-week treatment cycle. The most common adverse effects are fatigue, diarrhea, hand-foot syndrome, skin discoloration and hematological alterations (leukopenia, anemia and thrombocytopenia). The cardiovascular effects observed in these patients are hypertension (all grades 15% to 34%; grade 3: 4% to 13%), peripheral edema (24%), decline in ejection fraction (11% to 16%; grades 3/4: 1% to 3%), heart failure (≤ 15%) and chest pain (13%).^1^ Aortic dissection associated with sunitinib therapy is a rare adverse effect (< 1%).^2^ Aortic dissection generally occurs in patients with predisposing factors: hypertension, atherosclerosis, diabetes and Marfan’s syndrome.^3^ Hypertension is the main predisposing factor for aortic dissection but, in the medical literature, there is only one case of aortic dissection relating to sunitinib (Table 1).^4^ Here, we discuss the case of a 68-year-old patient with metastatic renal cell cancer who presented aortic dissection without preexisting hypertension, during sunitinib therapy at the usual doses.

**Table 1.**
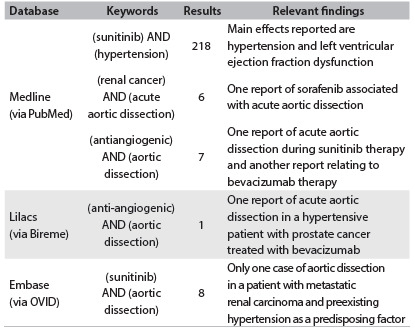
Search strategies used and results from each database

Last search in PubMed on January 12, 2014. Last search in Lilacs and Embase on September 20, 2013.

## CASE REPORT

Our patient was a 68-year-old man with a history of smoking (58 pack-years), who had undergone partial left nephrectomy in January 2006. Anatomopathological analysis showed a 25 mm clear cell carcinoma, of Fuhrman grade 2 and clinical stage T1N0M0. He was followed up with serial imaging studies.

In November 2009, bilateral renal and adrenal nodules were found on computed tomography scans. A biopsy was performed on the adrenal lesion. The result was consistent with metastatic clear cell carcinoma. His Memorial Sloan-Kettering Cancer Center score was low risk (Karnofsky performance score of 90%, with normal values for corrected serum calcium, lactate dehydrogenase and hemoglobin, and metastatic recurrence more than one year after the nephrectomy).

**Figure 1.**
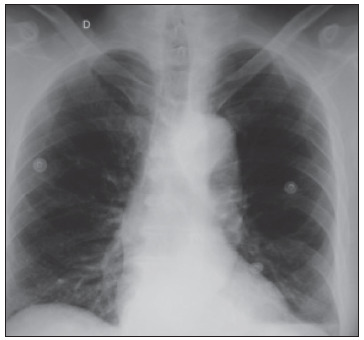
Chest X-ray showing enlargement of mediastinum.

**Figure 2.**
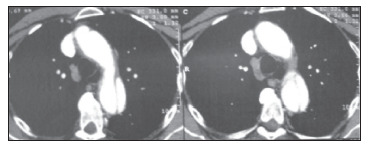
Chest computed tomography scan showing a false lumen in aorta (aortic dissection).

In April 2010, after clinical evaluation showing normal blood pressure levels, he was started on target therapy with sunitinib, at a daily dose of 50 mg (four weeks on and two weeks off). After the first cycle, his clinically evaluated blood pressure was normal. He began the second cycle and 20 days later, when he sought the emergency room with chest pain, his blood pressure was 210 mmHg x 120 mmHg. Electrocardiography did not show ischemic signals but the chest X-ray showed an enlarged mediastinum (Figure 1) and chest CT-angiography revealed Stanford type B aortic dissection from the aortic arch to the abdominal aorta (Figure 2). He received intravenous β-blocker (metoprolol) and sodium nitroprusside, and then the blood pressure dropped to 160 mmHg x 110 mmHg.

The patient was referred to a cardiovascular hospital, and an endoprosthesis was placed. He recovered well and started new therapy with temsirolimus, 25 mg weekly. His hypertension was managed using oral antihypertensives. After 10 weeks of therapy, the patient achieved stable disease, according to the Response Evaluation Criteria in Solid Tumors (RECIST) on computed tomography scans.

## DISCUSSION

The management of metastatic kidney cancer has changed over the last few years, with the advent of tyrosine kinase inhibitors such as sunitinib and sorafenib, the monoclonal antibody bevacizumab plus interferon, the antiangiogenic agent pazopanib and the mammalian target of rapamycin (mTOR) pathway inhibitors everolimus and temsirolimus. The main target of the therapy is inhibition of angiogenesis.^5^ The rapid growth of targeted therapies has raised the challenge of management of side effects. The main side effects relating to tyrosine kinase inhibitors are fatigue, gastrointestinal alterations and hypertension.

Hypertension occurs due to the effect of the drug on vascular endothelial growth factor (VEGFR) signal pathways by increasing systemic vascular resistance. The underlying pathophysiological mechanism is not completely known. The endothelial dysfunction reduces the density of microvessels through lowering the production of nitric oxide and increasing oxidative stress.^6^

Only one case of aortic dissection with sunitinib had previously been reported. In that report, a 58-year-old man with preexisting hypertension presented chest pain after four cycles of sunitinib. A computed tomography scan revealed descending aortic dissection. The drug was withdrawn and the antihypertensive therapy was modified.^4^ Acute aortic dissection relating to therapy with sorafenib, another antiangiogenic drug, and chemotherapy consisting of capecitabine and gemcitabine, in a 77-year-old patient with metastatic renal cell carcinoma, has been reported. In that case, the association between sorafenib and aortic dissection could not be affirmed because the chemotherapy might have played a role.^7^ Also, there is another report of aortic dissection associated with an antiangiogenic drug, in a 70-year-old patient with metastatic prostate cancer. Differing from our patient, he had preexisting hypertension, which gradually worsened with continued use of bevacizumab, a monoclonal antibody that acts as an angiogenesis inhibitor.^8^

The hypertensive crisis with aortic dissection that we observed in our patient without previous hypertension was a serious side effect. It was managed by means of drug withdrawal and treatment of the aortic dissection in accordance with cardiological guidelines.

Although our patient had a low-risk Memorial Sloan-Kettering Cancer Center score and clear cell carcinoma, we proposed to use temsirolimus on a trial basis, given that this drug has another mechanism of action and the rate of hypertension produced is very low. Mammalian target of rapamycin inhibitors are generally well tolerated and have low incidence of adverse events. The main concerns are stomatitis, hyperlipidemia, hyperglycemia and, rarely, non-infectious pneumonitis.^9^ Hypertensive crises relating to major vascular disorders such as aortic dissection and use of antiangiogenic drugs therapy can be correlated with these events.

## CONCLUSION

This report emphasizes the need for strict blood pressure control among patients taking antiangiogenic drugs. Blood pressure evaluations should be scheduled more frequently, especially at the beginning of the treatment, so as to enable early recognition and manage hypertension or other side effects.
